# Characterization of a hepatitis C virus genotype 1 divergent isolate from an HIV-1 coinfected individual in Germany assigned to a new subtype 1o

**DOI:** 10.1186/s12985-019-1135-7

**Published:** 2019-03-04

**Authors:** Bo Wang, Luise Krüger, Patrycja Machnowska, Amare Eshetu, Barbara Gunsenheimer-Bartmeyer, Viviane Bremer, Andrea Hauser, Norbert Bannert, C.-Thomas Bock

**Affiliations:** 1 Division of Viral Gastroenteritis, Hepatitis Pathogens and Enteroviruses, Department of Infectious Diseases, Robert Koch Institute, 13353 Berlin, Germany; 2 Division of HIV and other Retroviruses, Department of Infectious Diseases, Robert Koch Institute, 13353 Berlin, Germany; 3 Division of HIV/AIDS, STI and Blood-borne Infections, Department of Infectious Diseases Epidemiology, Robert Koch Institute, 13353 Berlin, Germany; 4 Institute of Medical Virology, Charité – Universitätsmedizin Berlin, 10117 Berlin, Germany; 5 Institute of Tropical Medicine, University of Tübingen, 72076 Tübingen, Germany

**Keywords:** Hepatitis C virus, HIV-1, HCV genotype 1 subtypes, Full-length genome, HVR1, RASs

## Abstract

**Background:**

HCV exhibits a high genetic diversity and is classified into 7 genotypes which are further divided into 86 confirmed subtypes. However, there are multiple isolates with unassigned subtypes. We aimed to amplify and characterize the full-length genome sequence of an HCV genotype 1 (HCV-1) divergent isolate (DE/17–0414) in Germany.

**Methods:**

The HCV infection was detected in an HIV-1-positive German female within an HCV/HIV-coinfection study using a commercially available antigen-antibody HCV ELISA kit and confirmed by an in-house quantitative real-time RT-PCR assay. Preliminary genotyping was done by sequencing and phylogenetic analysis on partial NS5B region. The full-length genome sequence was determined by consensus RT-PCR assays. Resistance-associated substitutions (RASs) were analyzed using the web-based tool Geno2pheno_[HCV]_.

**Results:**

Partial NS5B region of the isolate DE/17–0414 showed more than 95% identity to 73–08460349-1 l and HCV_Fr_003 from France and QC316 from Canada. Full-length genome analysis of the DE/17–0414 strain showed 91.8% identity to QC316 but less than 79.6% to other HCV-1 strains. Phylogenetic analyses demonstrated that DE/17–0414, 73–08460349-1 l, HCV_Fr_003, and QC316 formed a separate subcluster within HCV-1. DE/17–0414 had a distinct 3 amino acids insertion at the N-terminal of hypervariable region 1 (HVR1) within viral envelope glycoprotein 2 (E2) and several potential antiviral RASs among the NS3 and NS5A genes.

**Conclusions:**

We identified and analyzed an HCV-1 divergent isolate derived from an HIV-1 coinfected individual in Germany, which will be assigned to a new HCV-subtype 1o. Our understanding of the origin and transmission dynamics of this new subtype 1o requires further assessments from patients worldwide.

## Main text

Hepatitis C virus (HCV) causes both acute and chronic hepatitis. According to the World Health Organization (WHO), in 2015 an estimated number of 71 million people have been chronically HCV-infected globally [1]. Among these, approximately 4 to 5 million individuals are coinfected with HIV [2]. HCV/HIV-coinfections are of major public health concern, as HIV-coinfection is associated with sometimes more serious progression of HCV-infection [2]. Since 2011, direct-acting antivirals (DAAs) for various genotypes of HCV are available for standard-of-care treatment. However, there is a controversial discussion whether HIV-coinfection is associated with worse response to DAA-based therapy against chronic hepatitis C in real life than HCV-monoinfected patients [3, 4] and the occurrence of potential HCV resistance-associated substitutions (RASs) is correlated with treatment failure [5]. Therefore, detection of HIV/HCV-coinfection and monitoring of potential HCV RASs is of clinical importance [6]. HCV is a positive-strand RNA virus with a 9.7 kb single-stranded, messenger-sense RNA genome. HCV exhibits a high genetic diversity; there are 7 genotypes, further sub-divided into 86 confirmed subtypes according to the 10th International Committee on Taxonomy of Viruses (ICTV) report on the taxonomy of the family *Flaviviridae* [7]. Nonetheless, a number of HCV strains are phylogenetically divergent from previously described sequences, thus can only be classified into genotypes but without subtype assignment [8]. Globally, HCV genotype 1 (HCV-1) is dominant (46.2%) and different genotype/subtype prevalence evolves and correlates to epidemiological factors [9]. In Germany, a recent study reported that HCV-1a (35.9%) and HCV-1b (30.6%) are the most prevalent subtypes, followed by HCV-3 (20.6%) [10].

In this work, we aimed to amplify and characterize the full-length genome sequence of a HCV-1 divergent strain (DE/17–0414) from an HIV-1 coinfected individual from Germany*.* According to the “Protection against Infection Act” (IfSG; §7) diagnostic laboratories in Germany report new HIV infections anonymously to the Robert Koch Institute (RKI). Approximately 60% of the reports are submitted together with a dried serum spot (DSS) sample prepared from residual blood of the diagnosis. Antibodies and viral RNAs are isolated from these DSS and are used for sentinel studies (according IfSG §13) [11]. Within a sentinel study established at the RKI, HIV/HCV coinfections are analyzed. This includes partial sequencing for the determination of the HCV genotype. HCV-infection was serologically identified using the Monolisa HCV Ag/Ab ULTRA V2 kit (Bio-Rad, Marnes-la-Coquette, France). Viral RNA from DSS was extracted by the automated Nuclisens EasyMag platform (bioMerieux, Capronne, France) following the manufacturer’s instructions. HCV viral load was measured by an in-house quantitative RT-PCR assay targeting the 5′ noncoding region (Table 1). Preliminary HCV genotyping was done by a consensus nested RT-PCR assay targeting a 674 base pair fragment in the NS5B region corresponding to nt position 7962 to 8636 of H77 reference strain. After cDNA synthesis using the Transcriptor first-strand cDNA synthesis kit (Roche Diagnostics, Mannheim, Germany), the complete viral genome was amplified using KAPA HiFi HotStart ReadyMix PCR kit (Kapa Biosystems, Boston, USA) with HCV-1 degenerate and DE/17–0414 specific primers (Table 1). The 5′ and 3′ sequences were determined using 5′ and 3′ rapid amplification of cDNA ends (Roche Diagnostics, Mannheim, Germany). HCV amplicons were sequenced with the BigDye Terminator version 3.1 cycle sequencing kit (Applied Biosystems, California, USA) in both directions. The sequencing chromatograms were checked for overlapping multicolor peaks. Whole-genome sequence was assembled using Geneious software version 10.0.5 (Biomatters, Auckland, New Zealand) [12]. Sequence identity comparisons were performed using the BLASTn search engine (https://blast.ncbi.nlm.nih.gov). Phylogenetic analyses were completed using the Neighbor-Joining method with maximum composite likelihood nucleotide distance between coding regions and complete deletion option in MEGA software version 7 [13], Bootstrapping was performed with 1000 replicates. To identify possible recombination, identity plot and bootscan analyses of full-length sequences were performed in the SimPlot software program version 3.5.1 with a sliding window size of 300 nt and a step size of 15 nt increment [14]. Potential RASs analysis among NS3, NS5A, and NS5B regions were conducted by Geno2pheno_[HCV]_ – a web-based interpretation system [15]. Relative numbering of nucleotide (nt), amino acid (aa), insertions and deletions used the HCV reference isolate H77 (GenBank accession number AF009606) [16].

**Table 1:** Primers used for HCV quantification, genotyping and DE/17–0414 genome amplification

Primer^a^	Sequence (5′-3′)	Location^b^	Reference
Real-time RT-PCR assay for HCV quantification			
HCV-238_f	GAGGAACTACTGTCTTCACG	49–68	This study
HCV-239_r	TCGCAAGCACCCTATCAG	310–293	
HCV-240_f	TCGCAAGCACCCTATCAG	76–94	
HCV-235_r	AGTACCACAAGGCCTTTCG	290–272	
Heminested RT-PCR assay for HCV genotyping			
HCV-271_f	ACCACATCMRSTCCGTGTGG	7951–7970	This study
HCV-272_f	TCCGTGTGGRARGACYTSCTRGA	7962–7984	
HCV-305_f	CTCCGTMTGGGAGGACTTGC	7961–7980	
HCV-275_r	CTSGTCATAGCYTCCGTGAA	8635–8616	
Heminested RT-PCR assays for DE/17–0414 genome amplification			
HCV-235_r	AGTACCACAAGGCCTTTCG	290–272	This study
HCV-239_r	TCGCAAGCACCCTATCAG	310–293	
HCV-365_f	GGCGTTAGTATGAGTGTTGTGC	87–108	(Lu et al., 2014)
HCV-366_r	TCCCTGAAGAGTTGCGTATTCC	939–918	
HCV-367_r	AGAAAGAGCAACCGGGAAGATT	864–843	
HCV-368_f	TCTATCTTCCTTCTTGCCATCCTG	864–887	
HCV-369_f	AGGGATTTACCATGTCACCAATGA	935–958	
HCV-370_r	TCAAAGTCAGTAAGAGGTCGACAG	1747–1724	
HCV-371_f	CCCGGTGCATGGTAGACTAC	2164–2183	
HCV-372_r	CTCCACCCTCCGTTGGTTAG	3421–3492	
HCV-373_r	CCGTTGGTTAGGGAGTCAGC	3412–3393	
HCV-374_f	ACATTCTTGGCTACGTGCTGTA	3552–3573	
HCV-375_f	CCCCATTATCCAGATGTACACCAA	3635–3658	
HCV-387_r	TCTGGACTTCTCCCTCCACC	3531–3512	This study
HCV-388_f	GCCGCATCCAAACATTGAGG	4421–4440	
HCV-389_f	CGGCAAAGCTATCCCCCTAG	4478–4497	
HCV-390_r	CCCGCCTGTTTTGTCTGAGA	5089–5070	
HCV-391_f	GCATCCAAAGAGGCTGAGGT	5565–5584	
HCV-392_f	CATCCCTGCTGTCCCAACTT	5588–5607	
HCV-393_r	TTATGTCAGCTCCGCATGGG	6456–6437	
HCV-394_f	GACGCCGACCTCATAGAAGC	7017–7036	
HCV-395_r	TGGCGTAACAAGGAGTTGCT	7708–7689	
HCV-396_r	ATGGGCAGCTTGTTCTCCTC	7678–7659	
HCV-360_f	CTCACCTGCTATCTCAAGGCAA	8487–8508	
HCV-361_f	GTTATCTGTGAGAGTAGCGGGG	8574–8595	

^a^Forward primer designation end with _f; reverse primer designations end with _r

^b^Numbering is according to the HCV prototype strain of H77 (GenBank Acc. No. AF009606)

**Fig. 1:**
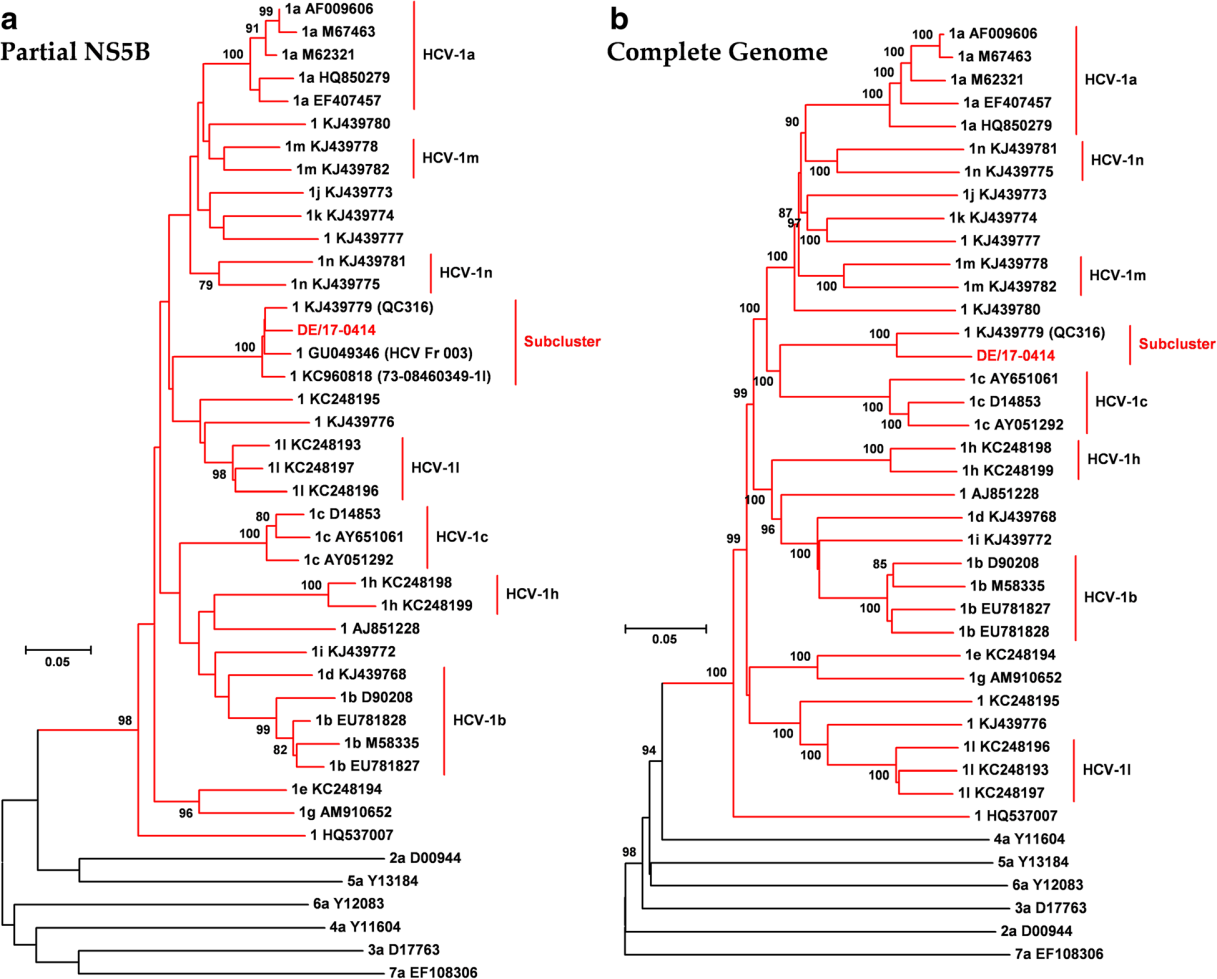
Phylogenetic relationships of DE/17–0414. The strain designations are indicated with geno/subtype and accession number at each branch. Clades corresponding to each genotype were supported by 100% of bootstrap replicates. Bootstrap values (> 75%) are indicated at specific nodes. Scale bars indicate the number of nt substitutions per site. HCV-1 subtypes and the new distinct sub-cluster are indicated on the right. DE/17–0414 of this study is highlighted in bold and red. (**a**) Phylogenetic analysis of representative HCV-1 strains based on 328 nt of partial NS5B sequences corresponding to nt positions 8283 to 8610 of H77 reference strain. (**b**) Phylogenetic analysis of HCV-1 complete coding region sequences

**Fig. 2:**
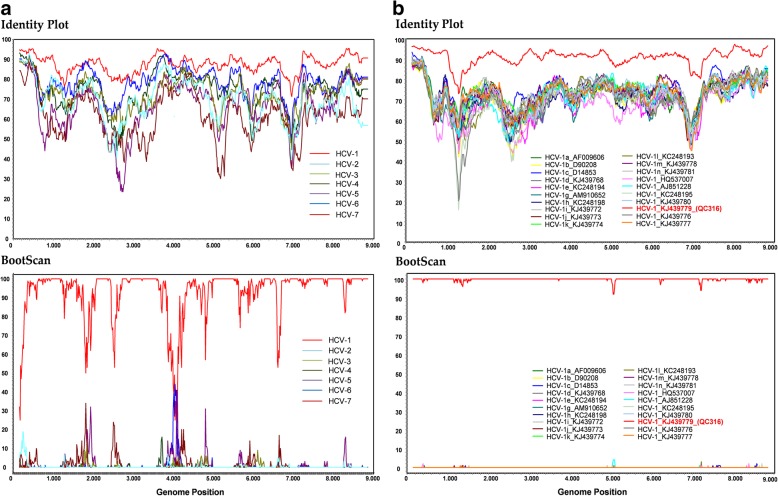
Analysis of potential recombination events of the DE/17–0414. Identity Plot and BootScan analyses of (**a**) HCV genotypes and (**b**) HCV-1 subtypes. All analyses were performed with a window of 300 nt and a step size of 15 nt under Kimura 2-parameter model. Positions containing gaps were stripped from the alignment. QC316 is highlighted in bold and red

**Fig. 3:**
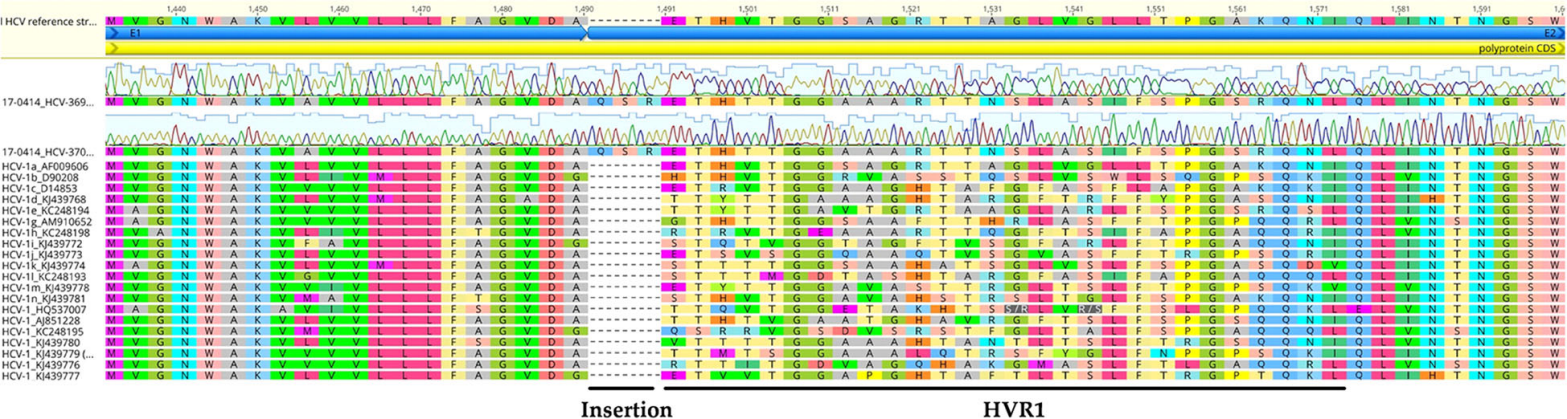
Sequence alignment of HCV-1 E1 and E2 genomic regions. The newly detected Q-S-R insertion of DE/17–0414 and HVR1 at the N-terminal of E2 is indicated at the bottom. Absolute numbering is corresponding to aa position 364 to 420 of H77 reference strain

A 63-year-old German heterosexual female, diagnosed with HIV-1 in 2017, was serologically positive for antigen/antibody combination HCV test. Viral load was 1.6 × 10^6^ IU/ml of DSS specimen. Preliminary sequence analysis based on partial NS5B sequences demonstrated that DE/17–0414 has an identity of 96.3% to the isolate QC316 (GenBank accession number KJ439779) from a Canadian immigrant with an origin in Cameroon [17]. It also shows high identities of 95.7 and 95.3% to isolates 73–08460349-1 l (GenBank accession number KC960818) and HCV_Fr_003 from France (GenBank accession number GU049346), respectively [18]. However, DE/17–0414 showed less than 83.6% identity to other HCV-1 strains. Phylogenetic analysis of representative HCV-1 to HCV-7 members of partial NS5B region suggested that DE/17–0414 belonged to HCV-1 forming an independent sub-cluster with HCV_Fr_003, 73–08460349-1 l, and QC316 (Fig. 1a). For a more comprehensive analysis of viruses belonging to the cluster, the full-length genome sequence of DE/17–0414 was amplified and sequenced. The complete genome of DE/17–0414 consisted of 9359 nt excluding the polypyrimidine tract, with a G + C content of 57.9% harboring the 10 HCV prospective genomic regions described in Table 2. The complete genome sequence of DE/17–0414 has been deposited in GenBank under the accession number MH885469. DE/17–0414 had the highest identity with the QC316 (91.8%) and less than 79.6% with any other HCV strain. Phylogenetic reconstructions based on the whole-genome sequences of HCV-1 strains showed that DE/17–0414 and QC316 formed to a separate subcluster within HCV-1 (Fig. 1b). Identity plot and bootscan analyses reflected no evidence for recombination between different HCV genotypes or HCV-1 subtypes (Fig. 2a and b). Intriguingly, a unique insertion of three aa (Q-S-R) was found at the N-terminal of hypervariable region 1 (HVR1) within viral envelope protein 2 (E2) (Fig. 3). In addition, several HCV-1 potential DAAs RASs including 36 L, 170 V (NS3 region) and 28 M, 31 M, 93H (NS5A region), were detected in DE/17–0414 (Table 3).

**Table 2:** Genomic regions of DE/−17–0414

Genomic region	NA numbering	AA numbering
5′ UTR	1–283	NA^a^
Core	284–856	1–191
E1	857–1432	192–473
E2	1433–2530	474–749
p7	2531–2719	750–812
NS2	2720–3370	813–1029
NS3	3371–5263	1030–1660
NS4A	5264–5525	1661–1714
NS4B	5526–6208	1715–1975
NS5A	6209–7552	1976–2423
NS5B	7553–9328	2424–3014
3′ UTR	9329–9359	NA

^a^NA for not applicable

**Table 3:** Insertion and potential direct-acting antivirals resistance-associated substitutions of DE/−17–0414

Genomic regions	Amino acid position	Reference amino acid	DE/−17–0414	Susceptibility to DAA according to Geno2Pheno[HCV] (Kalaghatgi et al., 2016)
E2	1a-1c	–	QSR	NA^a^
NS3	36	V	L	Substitution on scored position to Asunaprevir, Grazoprevir, Ledipasvir, Paritaprevir; Reduced susceptibility to Simeprevir, Telaprevir, Voxilaprevir; Resistant to Boceprevir
NS3	170	I	V	Substitution on scored position to Voxilaprevir
NS5A	28	L	M	Substitution on scored position to Ombitasvir
NS5A	31	L	M	Substitution on scored position to Velpatasvir
NS5A	93	Y	H	Substitution on scored position to Pibrentasvir; Resistant to Daclatasvir, Elbasvir, Ledipasvir, Ombitasvir, Velpatasvir

^a^NA for not available

The assignment of HCV into subtypes and genotypes is based on isolates that differ by 15–25% and by ≥30%, respectively, over their complete coding region sequence [8]. Both DE/17–0414 and QC316 exhibited close to 20% identity to other known HCV sequences. According to the ICTV criteria required for a new HCV genotype or subtype assignment which are: (1) one or more complete coding region sequence(s); (2) a distinct phylogenetic group from previously described sequences; (3) at least three epidemiologically unlinked isolates and (4) exclusion of intergenotypic or intersubtypic recombination [8]. The sequences from a total of 4 epidemiologically unlinked isolates that show more than 95% nucleotide identity have been identified for which the complete genome sequence is available for two of these and the partial NS5B sequence for the remaining two. Thus, this meets the criteria for the assignment of a new HCV subtype 1o. Subsequently, both DE/17–0414 and QC316 regarded as HCV-1o reference sequences.

The main observed genotypes/subtypes in Germany are HCV-1a, 1b and 3 [10]. In contrast, genetic diversity and distribution of other genotypes/subtypes are poorly documented. However, it is known that shifts or relative frequencies of HCV subtypes occurred in the last decades and the approval of DAAs for HCV-treatment is an additional factor, which will probably influence the subtype distribution [19]. Therefore, the knowledge on the genetic diversity of HCV is not only of epidemiological but also clinical significance. The core protein and envelope glycoproteins 1 and 2 constitute the structural elements of HCV [20]. The N-terminal of E2, called HVR1, is most divergent among HCV isolates and contributes to immune escape [21]. A distinct 3 aa (Q-S-R) insertion at the N-terminal of HVR1 was found in DE/17–0414 which exists in none of other known HCV strains. Whether the insertion is associated to HIV-coinfection and its function needs to be further analyzed. With the approval of DAA regimens testing HCV for RASs is clinically relevant. Several potential RASs were detected in the NS3 and NS5A genomic regions of DE/17–0414 on the basis of HCV subtypes 1a and 1b [6], indicating that corresponding DAAs should be avoided in this individual.

In conclusion, we identified and analyzed an HCV-1 divergent isolate from an HIV-1 coinfected individual in Germany, which will be assigned to a new subtype 1o with other three epidemiologically unrelated analogous HCV isolates. The origin and transmission dynamics of this new subtype needs further verification by more comprehensive genetic analyses of HCV strains from patients worldwide.

## Abbreviations

aa: Amino acid
DAAs: direct-acting antivirals
DSS: Dried serum spot
E2: envelope glycoprotein 2
HCV: Hepatitis C virus
HCV-1: Hepatitis C virus genotype 1
HIV-1: Human immunodeficiency virus type 1
HVR1: Hypervariable region 1
ICTV: International committee on taxonomy of viruses
nt: Nucleotide
RASs: Resistance-associated substitutions
RKI: Robert koch institute
WHO: World health organization

## References

[1] WHO: Hepatitis C Fact Sheet. Available online: http://www.who.int/news-room/fact-sheets/detail/hepatitis-c (accessed 22 October 2018).

[2] Operskalski EA, Kovacs A (2011). HIV/HCV co-infection: pathogenesis, clinical complications, treatment, and new therapeutic technologies. Curr HIV/AIDS Rep.

[3] Neukam K, Morano-Amado LE, Rivero-Juarez A, Mancebo M, Granados R, Tellez F, Collado A, Rios MJ, de Los Santos-Gil I, Reus-Banuls S (2017). HIV-coinfected patients respond worse to direct-acting antiviral-based therapy against chronic hepatitis C in real life than HCV-monoinfected individuals: a prospective cohort study. HIV Clin Trials.

[4] Schlabe S, Rockstroh JK (2018). Advances in the treatment of HIV/HCV coinfection in adults. Expert Opin Pharmacother.

[5] Sarrazin C (2016). The importance of resistance to direct antiviral drugs in HCV infection in clinical practice. J Hepatol.

[6] Pawlotsky JM (2016). Hepatitis C virus resistance to direct-acting antiviral drugs in interferon-free regimens. Gastroenterology.

[7] Simmonds P, Becher P, Bukh J, Gould EA, Meyers G, Monath T, Muerhoff S, Pletnev A, Rico-Hesse R, Smith DB (2017). ICTV virus taxonomy profile: Flaviviridae. J Gen Virol.

[8] Smith DB, Bukh J, Kuiken C, Muerhoff AS, Rice CM, Stapleton JT, Simmonds P (2014). Expanded classification of hepatitis C virus into 7 genotypes and 67 subtypes: updated criteria and genotype assignment web resource. Hepatology.

[9] Messina JP, Humphreys I, Flaxman A, Brown A, Cooke GS, Pybus OG, Barnes E (2015). Global distribution and prevalence of hepatitis C virus genotypes. Hepatology.

[10] Kartashev V, Doring M, Nieto L, Coletta E, Kaiser R, Sierra S, Group HCVES (2016). New findings in HCV genotype distribution in selected west European, Russian and Israeli regions. J Clin Virol.

[11] Hauser A, Hofmann A, Hanke K, Bremer V, Bartmeyer B, Kuecherer C, Bannert N: National molecular surveillance of recently acquired HIV infections in Germany, 2013 to 2014**.** Euro Surveill 2017, 22(2), pii: 30436. 10.2807/1560-7917.ES.2017.22.2.30436 PMC5404484 28105988

[12] Wang B, Yang XL, Li W, Zhu Y, Ge XY, Zhang LB, Zhang YZ, Bock CT, Shi ZL (2017). Detection and genome characterization of four novel bat hepadnaviruses and a hepevirus in China. Virol J.

[13] Kumar S, Stecher G, Tamura K (2016). MEGA7: molecular evolutionary genetics analysis version 7.0 for bigger datasets. Mol Biol Evol.

[14] Lole KS, Bollinger RC, Paranjape RS, Gadkari D, Kulkarni SS, Novak NG, Ingersoll R, Sheppard HW, Ray SC (1999). Full-length human immunodeficiency virus type 1 genomes from subtype C-infected seroconverters in India, with evidence of intersubtype recombination. J Virol.

[15] Kalaghatgi P, Sikorski AM, Knops E, Rupp D, Sierra S, Heger E, Neumann-Fraune M, Beggel B, Walker A, Timm J (2016). Geno2pheno[HCV] – a web-based interpretation system to support hepatitis C treatment decisions in the era of direct-acting antiviral agents. PLoS One.

[16] Kuiken C, Combet C, Bukh J, Shin IT, Deleage G, Mizokami M, Richardson R, Sablon E, Yusim K, Pawlotsky JM (2006). A comprehensive system for consistent numbering of HCV sequences, proteins and epitopes. Hepatology.

[17] Lu L, Li C, Xu Y, Murphy DG (2014). Full-length genomes of 16 hepatitis C virus genotype 1 isolates representing subtypes 1c, 1d, 1e, 1g, 1h, 1i, 1j and 1k, and two new subtypes 1m and 1n, and four unclassified variants reveal ancestral relationships among subtypes. J Gen Virol.

[18] Koletzki D, Dumont S, Vermeiren H, Fevery B, De Smet P, Stuyver LJ (2010). Development and evaluation of an automated hepatitis C virus NS5B sequence-based subtyping assay. Clin Chem Lab Med.

[19] Schroter M, Zollner B, Schafer P, Reimer A, Muller M, Laufs R, Feucht HH (2002). Epidemiological dynamics of hepatitis C virus among 747 German individuals: new subtypes on the advance. J Clin Microbiol.

[20] Penin F, Dubuisson J, Rey FA, Moradpour D, Pawlotsky JM (2004). Structural biology of hepatitis C virus. Hepatology.

[21] Bankwitz D, Vieyres G, Hueging K, Bitzegeio J, Doepke M, Chhatwal P, Haid S, Catanese MT, Zeisel MB, Nicosia A (2014). Role of hypervariable region 1 for the interplay of hepatitis C virus with entry factors and lipoproteins. J Virol.

